# Triamcinolone acetate and triamcinolone-isotretinoin efficacy in oral lichen planus management: Original clinical study

**DOI:** 10.6026/973206300220201

**Published:** 2026-01-31

**Authors:** Anish Ashok Gupta, Vatchala Rani R.M, Prarthana G.A, Vaibhavi Mehta, Monika Tanwar, Santosh Kumar

**Affiliations:** 1 Department of Oral Pathology & Microbiology, People's Dental Academy, People's University, Bhopal, Madhya Pradesh, India; 2 Department of Oral pathology and Microbiology, Faculty of Dentistry, Jamia Millia Islamia, New Delhi, India; 3 Department of Oral medicine and Radiology, Rajarajeswari Dental College and Hospital, Bangalore, Karnataka, India; 4 Department of Oral Medicine and Radiology, K.M. Shah Dental College and Hospital, Sumandeep Vidhyapeeth Deemed to be University, Piparia, Waghodia, Vadodara, India; 5 Department of Oral and Maxillofacial Surgery, SGT University, Gurgaon, Haryana, India; 6 Department of Periodontology & Implantology, Karnavati University, Uvarsad, Gandhinagar, Ahmedabad, Gujarat, India

**Keywords:** Oral lichen planus, triamcinolone acetonide, isotretinoin, combination therapy, Thongprasom score, malignant potential

## Abstract

Oral lichen planus (OLP) is a long-term inflammatory mucocutaneous disease that has the potential of malignant transformation. Hence, it requires effective long-term therapy. It was a randomized controlled, double-blind trial comparing triamcinolone acetonide 0.1 per cent oral paste (TA) to TA once daily with oral isotretinoin 10mg daily (TA+ISO) in 70 cases (biopsy-established) of OLP of more than 12 weeks duration. The TA+ISO group showed better clinical outcomes as the means of Thongprasom scores reduced in the TA group (p<0.001). TA+ISO showed more pain reduction (VAS) at 12 weeks (p<0.001), improved Histopathological (74.3) TA+ISO (p=0.006) and reduced Lesion size (p<0.001). TA in combination with isotretinoin is very useful as combination therapy in OLP and clinical and histopathological results are improved.

## Background:

Oral lichen planus (OLP) is one of the primary chronic inflammatory mucocutaneous diseases of the oral cavity with a prevalence rate of 0.5-2.2 percent of the general population [1]. OLP is characterized by T-cell-mediated autoimmune destruction of basal keratinocytes, and it is clinically presented as reticular, atrophic, erosive, or plaque-like lesions, which can be extremely painful, burning, and lead to poor quality of life [2]. OLP is a potentially malignant disease according to the World Health Organization (WHO) with malignant transformation rates of between 0.5 to 2 percent in the five years of the follow-ups and long term management is therefore vigilant in the cases [3]. Triamcinolone acetonide, which is a powerful topical corticosteroid, continues to be the ultimate choice of OLP treatment because of its anti-inflammatory and immunosuppressive effects [4]. Nevertheless, topical steroids have some limitations such as failure to respond in 3040 percent of patients, recurrence of the disease when the treatment is discontinued, local side effects such as candidiasis and mucosal atrophy [5]. Also, prolonged steroid use has systemic issues especially in the patient who needs large quantities of steroid use or those who have numerous comorbid conditions [6]. Such therapeutic issues highlight the urgency of the need to adopt alternative or adjunctive treatment options that can maximize effectiveness and reduce their side effects.

Isotretinoin, an artificial retinoid, has shown good immunomodulatory and anti-inflammatory effects in other mucocutaneous diseases [7]. Its action consists of the regulation of keratinocyte differentiation, inhibition of T-cell activation, and the regulation of cytokine ratios such as the down-regulation of interleukin-6 and tumor necrosis factor-alpha levels [8]. A number of uncontrolled studies have found positive outcomes of using isotretinoin in recalcitrant OLP and lesion regression rates are higher than 60 percent [9]. The synergistic effect of corticosteroids and retinoids has been theorized to be due to their complementary effects: steroids are instant anti-inflammatory, whereas isotretinoin corrects the underlying keratinocyte dys-regulation and immune dys-regulation of OLP pathogenesis [10]. In spite of these theoretical benefits, there is still conspicuously missing robust clinical evidence, comparing combined treatment with TA and isotretinoin to the current treatment with steroids including monotherapy. In a 2020 systematic review, only two pilot studies (n<30) assessed this combination, and none had any control group or standardized outcome measure [11]. The superiority of this regimen has not been proven by any randomized controlled trial yet, and neither optimum dosing nor safety profiles and influence of the regimen on histopathological characteristics are known. Furthermore, combination therapy with its impact on the quality of life impairment in OLP and the likelihood of decreasing the risk of malignant transformation is not studied [12]. Therefore, it is of interest to compare efficacy and safety of triamcinolone acetate monotherapy versus triamcinolone acetate combined with oral isotretinoin in biopsy-confirmed oral lichen planus, evaluating clinical response, pain relief, histopathological changes, quality of life, and malignant transformation risk.

## Materials and Methods:

## Study design and setting:

This prospective, double-blind, randomized, parallel-group controlled trial was conducted at the Department of Oral Medicine and Radiology, between January 2021 and December 2023.

## Sample size calculation:

Sample size was calculated based on a pilot study of 20 patients that demonstrated a mean difference of 1.5 points in Thongprasom scores between groups with a pooled standard deviation of 1.2. Using G*Power 3.1.9.7, with α=0.05, power=0.80, and effect size=1.25, the minimum sample required was 28 patients per group. To account for 20% attrition, 70 patients were recruited (35 per group).

## Participant selection:

Seventy patients aged 18-65 years with newly diagnosed, symptomatic, biopsy-confirmed OLP were enrolled. Inclusion criteria comprised: clinical and histopathological confirmation according to WHO criteria; at least one erosive or atrophic lesion >1 cm^2^; visual analogue scale (VAS) pain score ≥4; no prior treatment within 4 weeks. Exclusion criteria included: pregnancy or lactation; liver dysfunction (ALT/AST >2x normal); renal impairment (creatinine >1.5 mg/dL); history of isotretinoin intolerance; oral candidiasis; immunosuppressive therapy; malignancy; and uncontrolled systemic diseases.

## Randomization and blinding:

Patients were randomized 1:1 using computer-generated block randomization (block size 4) into Group A (TA alone) and Group B (TA+ISO). Allocation concealment was maintained using sequentially numbered, opaque, sealed envelopes. Patients, examining clinicians, and outcome assessors were blinded to group assignment. A separate pharmacist dispensed medications in identical packaging.

## Intervention protocol:

Both groups received triamcinolone acetonide 0.1% oral paste (Kenacort, Bristol-Myers Squibb) applied topically to lesions thrice daily after meals for 12 weeks. Group B additionally received oral isotretinoin 10mg capsules (Cipla Ltd.) once daily with meals. All patients received oral hygiene instructions and were prescribed antifungal prophylaxis (clotrimazole mouthwash) to prevent steroid- induced candidiasis.

## Outcome measures:

Primary outcome was clinical improvement measured by Thongprasom clinical scoring system (0=no lesions to 5=severe erythema/ulceration) at baseline, 4, 8, and 12 weeks. Secondary outcomes included: VAS pain scores (0-10); lesional surface area measured using digital photography and ImageJ software; histopathological grading (WHO criteria: band-like lymphocytic infiltrate, saw-tooth rete ridges, dysplasia) at baseline and 12 weeks; quality of life assessed using Oral Health Impact Profile-14 (OHIP-14); and adverse event monitoring.

## Assessment procedures:

Clinical photographs were standardized using a Canon EOS 80D with ring flash at fixed distance and magnification. Biopsy specimens were obtained from representative sites at baseline and week 12 under local anesthesia, processed with haematoxylin-eosin staining, and graded by a blinded oral pathologist. Blood tests (liver function, renal function, lipid profile) were performed at baseline, 6 weeks, and 12 weeks.

## Statistical analysis:

Data were analyzed using SPSS version 26.0. Normality was assessed via Shapiro-Wilk test. Independent t-test compared continuous variables between groups, while paired t-test evaluated within-group changes. Chi-square test analyzed categorical variables. Repeated measures ANOVA assessed temporal changes in clinical scores. Pearson correlation examined relationships between glycaemic status and outcomes. Statistical significance was set at p<0.05. Results were expressed as Mean±SD or percentages.

## Results:

All 70 patients completed the 12-week study (35 per group). Baseline demographics and disease characteristics were comparable between groups (p>0.05). Mean age was 42.6±11.3 years in Group A and 44.1±10.8 years in Group B (p=0.584). Female predominance was noted in both groups (Group A: 60.0%, Group B: 65.7%, p=0.631). The erosive type was most common (Group A: 54.3%, Group B: 51.4%). Mean baseline Thongprasom scores were 4.2±0.8 and 4.3±0.7 (p=0.623), VAS scores were 7.4±1.1 and 7.6±1.2 (p=0.487), and lesion areas were 3.8±1.4 cm^2^ and 3.9±1.3 cm^2^ respectively (Table 1). Group B demonstrated significantly greater improvement in Thongprasom scores at all-time points (p<0.001). At 12 weeks, mean Thongprasom score was 1.1±0.6 in Group B versus 2.4±0.9 in Group A (mean difference 1.3, 95% CI: 0.9-1.7). VAS scores decreased progressively in both groups, with Group B achieving superior pain reduction: 1.3±0.8 at 12 weeks versus 3.1±1.0 in Group A (p<0.001). Repeated measures ANOVA confirmed significant timexgroup interactions for both Thongprasom score (F=34.67, p<0.001) and VAS (F=28.34, p<0.001) (Table 2). Lesion area reduction was significantly greater in Group B at all follow-ups. At 12 weeks, Group B achieved 68.4±12.3% reduction versus 41.2±15.6% in Group A (p<0.001). Complete clinical resolution (Thongprasom score ≤1) occurred in 17 (48.6%) Group B patients versus 7 (20.0%) Group A patients (p=0.011). Histopathological examination at 12 weeks showed improvement in 26 (74.3%) Group B patients compared to 15 (42.9%) Group A patients (p=0.006), with greater reduction in lymphocytic infiltrate density and basal cell degeneration. OHIP-14 scores improved significantly more in Group B (baseline 32.4±6.8 to 12.1±4.3) than Group A (31.8±7.2 to 18.7±5.9) (p<0.001) (Table 3). Adverse events were mild and self-limiting. Mucosal dryness occurred more frequently in Group B (40.0% vs 22.9%), as expected with isotretinoin. Transient hypertriglyceridemia was observed in 3 Group B patients, resolving without intervention. No patients discontinued treatment due to adverse effects. No clinically significant hepatotoxicity or renal dysfunction was detected.

## Discussion:

This randomized controlled trial indicates that combination therapy consisting of triamcinolone acetonide together with oral isotretinoin is significantly better than steroid monotherapy in the treatment of oral lichen planus in a variety of outcome domains. The higher clinical effect, improved analgesia, higher lesion healing, and histopathological improvement all prove the need to incorporate isotretinoin into the OLP treatment algorithms as an effective adjunct. The first outcome, Thongprasom clinical score, had found a 1.3-point better result in the combination group at 12 weeks. This variation is greater than the minimal important difference in clinical terms of 1.0 that is set regarding OLP severity scales [13], which validates actual clinical significance. Of special interest is the fact that the complete resolution rate in the TA+ISO group (48.6 percent) is much higher than that recorded with TA alone (20.0 percent) because complete remission has been challenging with traditional therapy [14]. The quick improvement in progress, which was observed during week 4, indicates the synergy and not just the additive effects between the agents. The decrease in pain measured by VAS showed a reduction of 2.8 points, which were significantly higher in the combination group. This is important to the clinic as pain is the most disabling symptom in OLP which significantly impairs mastication, speech, and quality of life [2]. The increased analgesia is probably due to a decreased inflammation (steroid effect) as well as a decrease in neuronal sensitization mediated by the effect of isotretinoin to modulate the inflammatory cytokines [15]. The correlation of clinical and pain scores (r=0.73, p<0.001) is an indication that the positive change in lesions is directly proportional to the reduction in symptoms.

The fact that improvement in histopathology in 74.3% of the combination group patients is a key finding. Malignancy of OLP is associated with lymphocytic infiltration and basal cell degeneration. Normalization of keratinocyte differentiation and decrease in dysplasia by isotretinoin might help in the mitigation of transformation risk [16]. Although the 12 weeks of follow-up we conducted do not allow conclusive risk cancer assessment, the reduction of the inflammatory infiltrate was significantly high and should be considered a possibility of a chemo preventive effect that needs to be monitored over a longer-time period. The mechanism of this synergism is by the use of complementary pathways. Triamcinolone acetonide inhibits T-cell, and pro- inflammatory cytokine production, which offers a fast control of symptoms [5]. Isotretinoin, the derivative of vitamin A, controls the epithelial differentiation with the participation of retinoic acid receptor and reverses hyperkeratotic and atrophic changes typical of OLP. Also, isotretinoin suppresses matrix metalloproteinases and interferon-gamma pathways, which is much more fundamental to the autoimmune etiology than steroids alone [7]. This two-pronged treatment of inflammation as well as epithelial dysregulation is likely the reason behind the better results. There is little quality evidence on comparative literature. Topical TA with our lower dose (10mg) had a greater effect and better tolerability. Other comparative trials on tacrolimus versus TA had reported similar response rates to ours in TA +ISO group but topical calcineurin inhibitors have carcinogenicity issues. The positive safety profile of our regimen, dryness of mild levels and reversible lipid alterations, makes it a feasible substitute of immunomodulators [17].

Patient-centered benefits are emphasized in the OHIP-14 quality of life improvement (reduced by 20.3 points in the combination group). OLP has a strong negative effect on the oral health-related quality of life, and the scores are associated with disease activity. The size of the improvement is bigger than with steroid-only trials, which indicates that improved clinical control can be translated to a significant restoration of life quality [18]. Negative incidents were treatable and anticipated. Our dose-selection strategy is justified by the lower incidence of hypertriglyceridemia with low-dose isotretinoin compared to the 1520 percent incidence with the standard 20-40mg doses of isotretinoin in dermatology. Mucosal dryness which was more common in the combination group was alleviated using lip balms and lubricating gels. It is interesting to note that oral candidiasis was less prevalent in the combination group (5.7% vs 14.3%), which might be explained by the antifungal effect of isotretinoin manifested by improvements in the quality of keratinocyte barrier [19]. Limitations of the study are a relatively brief period of a 12-week follow-up, which is unable to determine long-term rates of relapse and prevention of malignant transformation. The duration of a longitudinal study of 2-5 years is required to prove durability and safety of oncology. The single-center design can restrict external validity but our hard and fast diagnostic criteria improve internal validity. The omission of symptomatic reticular OLP may not be representative of the real practice in which the symptomatic cases are usually noted as opposed to being treated. Also, we had not quantified serum retinoids or immune markers to exclude mechanistic validation of drug action. The next studies should be carried out in terms of dose-response issues, possibly finding out the minimum doses of isotretinoin to use to prevent adverse outcomes even further. Relative efficacy would be made clear with comparative studies with other adjuncts (tacrolimus, apremilast). Personalized therapy could be achieved with the help of molecular profiling to identify patients with the highest potential of benefiting maybe high- grade dysplasia or certain cytokine profiles. Lastly cost-effectiveness studies comparing the use of combination therapy with sequential treatment plans would enlighten the healthcare policy.

## Conclusion:

The current randomized trial offers solid evidence that low-dose oral isotretinoin with triamcinolone acetonide is effective in improving clinical, histopathological and quality of life outcomes when compared to steroid monotherapy use in OLP. The risk-benefit profile enables its use as a first-line regimen in the symptomatic management of OLP, especially in those who have moderate-to-severe disease or those who are at greater risk of malignancy. Prolonged clinical trials are justified to verify long-term advantages and safety in the oncology.

## Figures and Tables

**Table 1:** Baseline demographic and clinical characteristics

**Characteristic**	**Group A (TA alone) (n=35)**	**Group B (TA+ISO) (n=35)**	**p-value**
Age (years), Mean±SD	42.6±11.3	44.1±10.8	0.584
Gender (female), n (%)	21 (60.0)	23 (65.7)	0.631
Disease duration (months), Mean±SD	8.4±3.2	9.1±3.8	0.418
Clinical type, n (%)			0.823
Reticular	6 (17.1)	7 (20.0)	
Erosive	19 (54.3)	18 (51.4)	
Atrophic	7 (20.0)	8 (22.9)	
Plaque-type	3 (8.6)	2 (5.7)	
Thongprasom score, Mean±SD	4.2±0.8	4.3±0.7	0.623
VAS pain score, Mean±SD	7.4±1.1	7.6±1.2	0.487
Lesion area (cm^2^), Mean±SD	3.8±1.4	3.9±1.3	0.719
SD: Standard Deviation;
VAS: Visual Analog Scale;
TA: Triamcinolone Acetonide;
ISO: Isotretinoin

**Table 2:** Clinical and pain outcome measures over 12 weeks

**Parameter**	**Group**	**Baseline**	**4 weeks**	**8 weeks**	**12 weeks**	p-value*
Thongprasom score	A	4.2±0.8	3.1±0.9	2.7±0.8	2.4±0.9	<0.001
	B	4.3±0.7	2.4±0.8	1.6±0.7	1.1±0.6	
VAS pain score	A	7.4±1.1	5.2±1.3	4.1±1.2	3.1±1.0	<0.001
	B	7.6±1.2	3.8±1.1	2.4±1.0	1.3±0.8	
Values are Mean±SD;
*p-value for timexgroup interaction;
VAS: Visual Analog Scale (0-10)

**Table 3:** Lesion area, histopathological, and quality of life outcomes

**Outcome**	**Group A (TA alone)**	**Group B (TA+ISO)**	**p-value**
Lesion area reduction at 12 weeks (%)	41.2±15.6	68.4±12.3	<0.001
Complete clinical resolution, n (%)	7 (20.0)	17 (48.6)	0.011
Histopathological improvement, n (%)	15 (42.9)	26 (74.3)	0.006
OHIP-14 score at baseline, mean±SD	31.8±7.2	32.4±6.8	0.712
OHIP-14 score at 12 weeks, mean±SD	18.7±5.9	12.1±4.3	<0.001
**Adverse events, n (%)**			
Mucosal dryness	8 (22.9)	14 (40.0)	0.102
Mild cheilitis	2 (5.7)	6 (17.1)	0.131
Transient hypertriglyceridemia	0 (0)	3 (8.6)	0.079
Oral candidiasis	5 (14.3)	2 (5.7)	0.227
OHIP-14: Oral Health Impact Profile-14 (range 0-56)
